# Demographic and emission drivers of household air pollution in biomass using communities

**DOI:** 10.1038/s41598-026-49847-8

**Published:** 2026-05-18

**Authors:** Seyed Hamed Godasiaei, Obuks A. Ejohwomu, Majeed Oladokun, Faris Elghaish

**Affiliations:** 1 School of Chemical Engineering and Technology, Xi’an Jiaotong University, Xi’an, China; 2 Department of Civil Engineering and Management, University of Manchester, Engineering Building A, Booth Street E, Manchester, M13 9PL UK; 3 CIDB Centre of Excellence, University of Johannesburg, Johannesburg, 2092 South Africa; 4 School of Natural and Built Environment, Queen’s University Belfast, Belfast, UK

**Keywords:** Cleaner cooking, Biomass fuels, Developing country, Explainable boosting machines (EBM), Graph neural networks (GNN), Recurrent neural networks (RNN), Environmental sciences, Environmental social sciences

## Abstract

This study investigates household air pollution from biomass combustion by integrating a household survey, experimental stove testing, and data-driven analysis. A structured survey of 1200 households captured socio-economic conditions, cooking practices, and health outcomes, showing that 57.4% rely on agriculture and 14.8% report health symptoms linked to traditional stove use, while 33.1% have adopted improved stoves. Experimental testing evaluated stove performance and emissions under real operating conditions. Results indicate that thermal efficiency peaks at 37% during the simmering phase and declines during cold and hot starts. Emissions were highest during cold start, with CO reaching 123 g/MJ and PM 1172 mg/MJ, but decreased by more than 40% during stable operation, particularly when charcoal was used. Temperature was identified as an important environmental factor, reducing CO and PM concentrations while increasing NO_2_ formation. Overall, the findings highlight the combined influence of technology performance, user practices, and environmental conditions on household air pollution. The study underscores the importance of promoting cleaner cooking technologies, improving stove operation, and integrating socio-economic considerations into intervention strategies to reduce health risks and support sustainable household energy transitions.

## Introduction

Household air pollution from incomplete biomass combustion continues to be a critical public health and environmental problem in low resource communities^1,2^. Biomass burning in traditional methods of cooking, such as three-stone fires and inefficient stoves, generates harmful pollutants, including fine particulate matter (PM_2.5_), carbon monoxide (CO), black carbon (BC)^3,4^. These pollutants not only degrade indoor environments but also have far-reaching environmental consequences. The reliance on biomass fuels accelerates deforestation, worsens outdoor air pollution, and contributes significantly to climate change^5,6^. Among the emissions, black carbon plays a particularly damaging role as a short-lived yet highly potent climate forcer, intensifying atmospheric warming and impeding global efforts to achieve net-zero emissions^7,8^. Additionally, adverse health effects are serious, from chronic obstructive pulmonary disease and respiratory infections to cardiovascular diseases, pregnancy complications, and child mortality^9,10^. The health risks are not borne equally but disproportionately affect vulnerable groups-especially women and children, who spend more time near cooking areas^11^. Despite ongoing intervention programs, which reduce household air pollution (HAP), the acceptance of clean cooking fuel alternatives such as liquefied petroleum gas (LPG), electricity, and solar stoves is extremely limited due to cost, infrastructure, and socio-cultural barriers^12,13^. Improved cookstoves are intermediate solutions that have been designed to reduce emissions and optimize fuel combustion^14,15^. However, many improved cookstove (ICS) models fall short in complying with practical users’ needs under real field conditions due to their limited design and performance, besides not being well adapted to traditional cooking practices^16^. Also, empirical evidence to support claims of pollutant reduction and field reliability is usually lacking^17^.

The impact of HAP from solid fuel consumption has been extensively studied for its health and environmental consequences. Lee et al.^18^ investigated the link between prolonged exposure to HAP and human depression, finding a significant association between long-term exposure to solid fuel use and an increased risk of depression in older Chinese adults. Specifically, HAP from burning wood for heating and cooking was a notable contributor to depression risk. Similarly, Ahmed et al.^19^ focused on indoor air quality’s effects on women’s health, emphasizing the critical role of cooking fuel type. Their study highlighted its influence on pregnancy outcomes, including the termination and duration of pregnancies, as well as the years and age at sterilization. They also identified cooking locations as a significant factor, further underscoring the broader health implications of household environmental conditions for women. Ali et al.^20^ explored the effects of indoor air pollution caused by household solid fuel use on women and children. Their findings revealed that pollutants such as polycyclic aromatic hydrocarbons, particulate matter, nitrogen oxides, carbon monoxide, and sulfur dioxide were present at concentrations two to three times higher indoors compared to outdoors. These pollutants induce toxic mechanisms, including oxidative stress, DNA methylation, and gene activation. For children, exposure from birth is associated with low birth weight, respiratory infections, anemia, and premature mortality. Women experience heightened risks of lung cancer, chronic obstructive pulmonary disease (COPD), and cardiovascular diseases, resulting in disability and premature death. Men et al.^21^ examined the adoption of separate kitchens and mechanical ventilation in rural areas. Their findings showed that ventilation fans and separate kitchen designs significantly reduce premature deaths. They emphasized that cost-effective kitchen renovations provide substantial health benefits and serve as a practical solution to challenges faced during the transition to clean energy in rural communities. In India, Rao et al.^22^ found that households contribute to ambient PM_2.5_ directly via biomass-fueled cooking and transport stoves, and indirectly through purchases driving manufacturing. Indirect PM_2.5_ emissions are nearly double direct emissions from cooking stoves. They suggested that pollution control measures at the industrial level could effectively reduce health inequalities caused by ambient air pollution. Ranzani et al.^23^ studied the relationship between ambient PM_2.5_, HAP, and lung function in young adults. Among 1,044 participants, with an average age of 22 years, 76% relied on biomass as their primary cooking fuel. Their findings showed a negative association, with biomass use linked to a reduction of -112 mL/min in lung function. The impact was more severe for users of unventilated stoves than for those using ventilated stoves, underscoring the detrimental effects of ambient PM_2.5_ and HAP on respiratory health, particularly in young populations. Additionally, research on improved cookstoves (ICS) has demonstrated their potential to reduce greenhouse gas emissions and improve fuel efficiency^24^. Roy and Acharya^25^ analysed energy inequality and air pollution in India, reporting a 15% increase in LPG consumption between 2015 and 2019. Despite this progress, over 60% of poorer households and more than 90% of the poorest households still rely on solid biomass for cooking. They found no significant correlation between LPG use and ambient PM_2.5_ levels (*r* = 0.036; p ≫ 0.05), indicating that other confounding factors may weaken the expected air quality benefits of clean fuel adoption. Furthermore, although the Pradhan Mantri Ujjwala Yojana (PMUY) has expanded LPG access, inadequate subsidies and persistently low consumption among poorer populations may hinder progress toward the World Health Organization’s air quality targets. Archer-Nicholls et al.^26^ assessed the contribution of household combustion to ambient air pollution and health impacts in China. Their modelled PM2.5 concentrations closely matched ground observations and attributed approximately 341,000 premature deaths (95% CI 306,000–370,000) to household combustion emissions. Of these, about 159,000 and 182,000 deaths were linked to heating and cooking emissions, respectively, representing nearly one-third of PM2.5-related mortality. Other research highlights the health risks associated with specific cooking practices. Wang et al.^27^ examined emissions from Chinese cooking methods, noting that high-temperature oil-based cooking can generate substantial pollutants. The highest PM concentrations (0.14–24.46 mg/cm^3^) occurred when wood or raw coal were used, high-temperature cooking was performed without emission controls, and olive oil was applied. VOC concentrations ranged from 0.35 to 3.41 mg/m^3^, with barbecues producing the highest levels. Gaseous pollutant emissions were greatest under inefficient combustion conditions (CO_2_/(CO + CO_2_) < 0.5). Similarly, Nduka et al.^28^ explored the relationship between cooking energy, indoor air pollution, and women’s well-being in Nigeria. Clean cooking energy use was strongly associated with improved happiness and life satisfaction, as well as reduced mental health challenges. In contrast, carbon monoxide exposure negatively affected women’s health and overall well-being. However, adoption remains low due to user perceptions of ICS being less effective or durable than traditional cookstoves (TCS) and three-stone fires (TSF), particularly in rural and culturally specific contexts^29^. This ongoing reliance on TCS and TSF poses significant health risks, especially for women and children, who are disproportionately affected by biofuel-related pollution^30,31^. Addressing these challenges remains an urgent priority, with further research and practical solutions needed to improve household air quality and reduce associated health risks globally^32,33^.

Household air pollution from incomplete biomass combustion remains a major environmental and public health challenge in many low- and middle-income regions, particularly in sub-Saharan Africa where reliance on solid fuels for cooking is widespread. In Kenya, and especially in Kericho County, biomass fuels such as firewood and charcoal continue to dominate household energy use due to affordability, cultural cooking practices, and limited access to cleaner alternatives. Traditional stove technologies, inefficient combustion, and poorly ventilated kitchens contribute to high indoor pollutant concentrations, while emissions released outdoors may further influence ambient air quality. However, the interaction between indoor and outdoor pollution, together with the role of household behaviour, stove usage patterns, and socio-economic conditions, remains insufficiently understood in this local context. Previous studies have examined the health and environmental impacts of household air pollution and the performance of improved cookstoves, yet important gaps remain. Many investigations rely on simplified empirical or linear approaches that do not fully capture the complex, nonlinear relationships between environmental conditions, combustion processes, and pollutant formation. Moreover, relatively few studies have integrated household characteristics, behavioural factors, and real-world field measurements to explain how and why pollution exposure varies across homes in rural African settings such as Kericho County. Recent advances in machine learning have shown strong potential for analysing complex environmental systems, including indoor and household air quality. These approaches can identify hidden patterns, model nonlinear interactions, and improve prediction of pollutant dynamics under varying environmental and behavioural conditions. Despite this potential, the application of interpretable machine learning to understand biomass stove emissions, indoor-outdoor air quality relationships, and household exposure in sub-Saharan Africa remains limited. To address these gaps, this study combines household survey data with field measurements to investigate the drivers and impacts of indoor air pollution in biomass-dependent homes in Kericho County, Kenya. Commonly, most traditional approaches toward HAP mitigation rely on either static or very simplified empirical models that cannot consider dynamic environmental conditions and user behavior. In this paper, a bridge is developed between empirical insights and adaptive solutions, enabling better stove design and more accurate pollutant emission predictions. Machine learning enables patterns and correlations that are often missed in conventional methods to provide tailored solutions addressing data-driven local needs and constraints.

## Materials and methods

### Integrating socioeconomic factors, field measurements, and experimental approaches

In Kericho County, Kenya, socioeconomic challenges combined with traditional cooking practices create a situation where households must often choose between affordable energy and safe living. The selection of cooking fuels and stoves is driven by financial limitations, cultural norms, and resource availability, all of which directly affect particulate matter emissions and associated health risks such as respiratory issues and eye irritation. These dynamics underscore the need for site-specific interventions to improve indoor air quality and public health in rural areas. Inequities in land ownership and education further complicate the adoption of clean cooking technologies, highlighting the importance of integrated environmental and social solutions. A baseline survey of 1200 households across Belgut, Kipkelion East, and Soin/Sigowet was conducted over ten days by 15 trained enumerators, with data carefully screened for analysis. In urban areas like Nairobi, HAP is further worsened by traffic and industrial emissions, making it harder to isolate cooking-related pollution. While experimental studies show emissions vary based on stove type, fuel, and ventilation, they often fail to capture broader socioeconomic and environmental influences. To fill this gap, calibrated sensors were deployed to measure PM_2.5_, PM_10_, CO, NO_2_, and O_3_, with outdoor sensors placed close together for consistent, validated readings. Indoor pollution from biomass stoves typically exceeds outdoor levels, and while ventilation helps, its effectiveness is limited when outdoor air is already polluted. Over 49 days, continuous monitoring tracked temperature, humidity, pressure, NO, NO_2_, O_3_, and PM (PM_1_, PM_2.5_, PM_10_). Fine particles like PM_2.5_ can deeply penetrate the lungs, causing cardiovascular and respiratory diseases, while NO and NO₂ contribute to smog and breathing difficulties. Ozone, a secondary pollutant formed from NOₓ and sunlight, further irritates the respiratory system. Environmental factors like temperature, humidity, and pressure affect pollutant behavior and accumulation (see Table 1).

**Table 1:** Air quality device specifications and environmental conditions for correlating outdoor and indoor pollution.

Measure	Estimated accuracy	Range	Limits of detection
Nitrogen dioxide (NO_2_)	10 µg/m^3^ (5.2 ppbV)	0–20,000 µg/m^3^ (0–10,000 ppbV)	1.5 µg/m^3^
Nitrogen oxide (NO)	10 µg/m^3^ (8 ppbV)	0–6,000 µg/m^3^ (0–5,000 ppbV)	1.5 µg/m^3^
Ozone (O_3_)	15 µg/m^3^ (7.5 ppbV)	0–15,000 µg/m^3^ (0–7,500 ppbV)	1.5 µg/m^3^
Particulate matter (PM_1_)	5 µg/m^3^	0–20,000 µg/m^3^	0.2 µg/m^3^
Particulate matter (PM_2.5_)	5 µg/m^3^	0–20,000 µg/m^3^	1.3 µg/m^3^
Particulate matter (PM_10_)	5 µg/m^3^	0–20,000 µg/m^3^	1.4 µg/m^3^
Carbon monoxide (CO)	0.3 mg/m^3^ (0.3 ppmV)	0–40 mg/m^3^ (0–35 ppmV)	0.03 mg/m^3^
Sulphur dioxide (SO_2_)	20 µg/m^3^ (7.6 ppbV)	0–6500 µg/m^3^ (0–2,500 ppbV)	1.5 µg/m^3^
Carbon dioxide (CO_2_)	30 ppmV	0–5000 ppm	405 ppmVb
Total organic volatile compounds (TVOCs).	–	0–15,000 ppbV	1 ppbV
Pressure	1.2 hPa	300–1,100 hPa	–
Temperature	≤ 1 °C	− 20 °C to + 45 °C	–
Relative humidity	5%	15–85%	–

### Effective techniques for data collection, feature extraction, and information preparation for ML algorithms

**Fig. 1:**
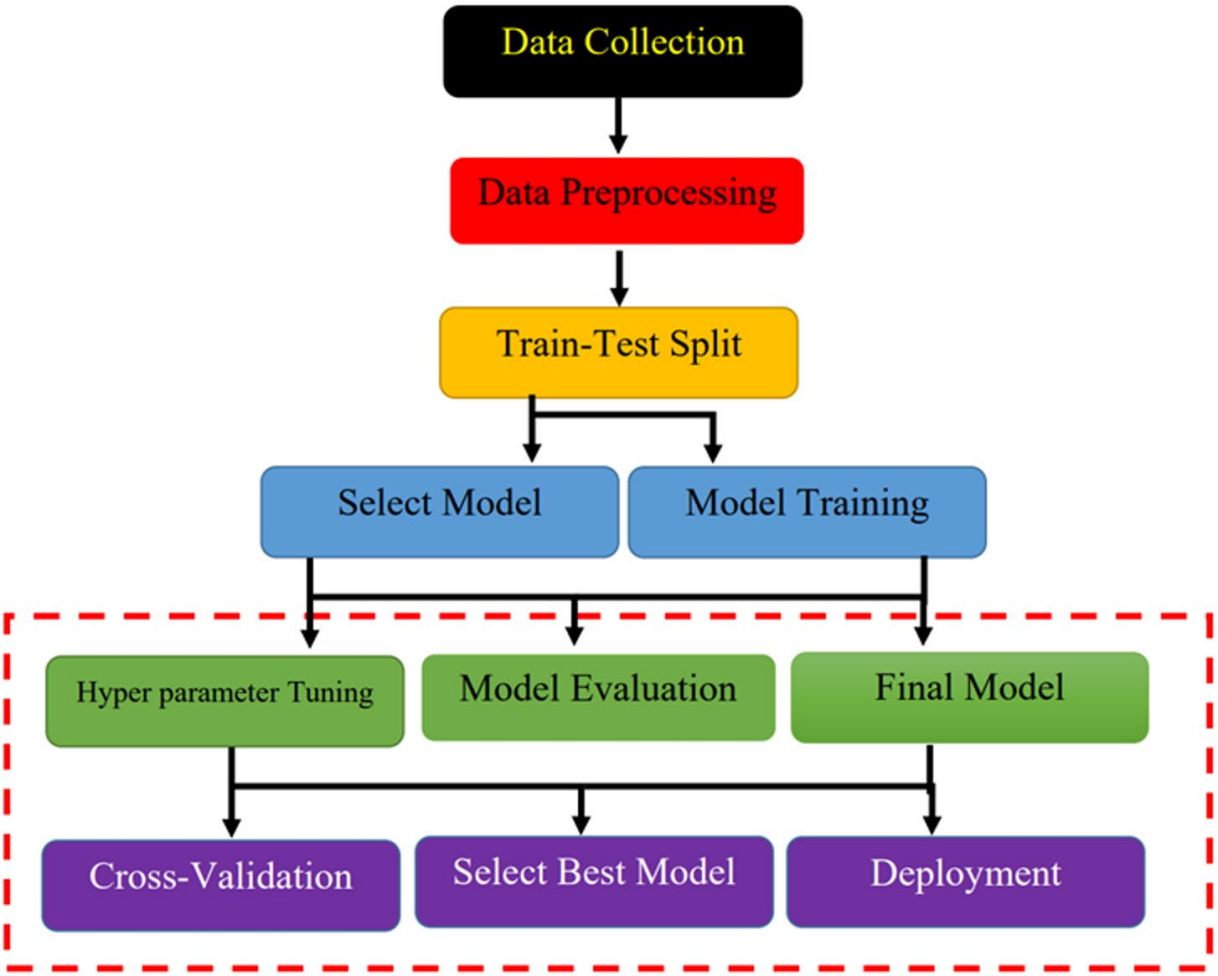
Flowchart of ML model training and optimization process.

Monitoring air quality and environmental conditions within households is essential for accurately assessing exposure risks and designing effective mitigation strategies. While conventional air quality monitoring depends on expensive reference-grade instruments, this study employed integrated low-cost sensors capable of measuring multiple pollutants, including PM_2.5_, CO, and NO_2_, to enable extensive field deployment in resource-limited settings. To ensure data quality and reliability, a rigorous multi-stage calibration protocol was implemented. Prior to deployment, all sensors were co-located with reference-grade instruments under controlled and real-world environmental conditions, where simultaneous measurements were collected across a wide range of pollutant concentrations. Calibration models were developed using regression-based correction techniques to minimize systematic bias and sensor drift, with temperature and relative humidity incorporated as covariates to account for environmental influences on sensor response. During field operation, periodic co-location checks, zero/span verification, and routine quality control procedures, including outlier detection, signal filtering, and drift adjustment, were conducted to maintain measurement stability. Following calibration, the sensors showed strong agreement with reference measurements, with PM_2.5_ typically achieving good coefficients of determination and significantly reduced error and bias. Comparable improvements were observed for CO and NO_2_, confirming that the calibration process substantially enhanced measurement accuracy and ensured the robustness of the dataset used for subsequent analysis. Building on this calibrated sensing framework, the study integrates advanced machine learning techniques (EBM, RNN, and GNN) to analyze the dynamic relationship between indoor and outdoor air pollution in households reliant on biomass fuels. These approaches enable detection of complex nonlinear patterns, prediction of pollutant concentrations, classification of air quality levels, and identification of key environmental drivers influencing pollution variability. Particular emphasis was placed on PM_2.5_ due to its well-established long-term health impacts, including chronic respiratory illness and persistent breathing difficulty associated with prolonged exposure. The analysis utilized a large experimental dataset comprising 529,462 records with nine input variables and one target variable, enabling robust modeling of temporal pollution behavior. RNN and GNN architectures were selected for their ability to capture time-dependent relationships, allowing deeper insight into how environmental and operational factors shape pollution dynamics. The dataset was divided into training (80%) and testing (20%) subsets, and model performance was optimized through hyperparameter tuning and cross-validation to prevent overfitting. All analyses were conducted in Python using the Scikit-learn framework, ensuring consistent training procedures, reproducibility, and reliable evaluation of predictive performance (Table 2, Fig. 1).

**Table 2:** The characteristics of the dataset utilized in this study’s input and output.

No.	Parameter			Unit	Subscript	Range
1	Temperature	Input		[°C]	T	12.6–31.8
2	Humidity	Input		[%]	H	22.3–88.5
3	Pressure	Input		[mbar]	P	79,953–82,126
4	Nitric oxide	Input		[ppb]	NO	0–753.35
5	Nitrogen dioxide	Input		[ppb]	NO_2_	0–1054.25
6	Ozone	Input		[ppb]	O_3_	0–391.51
7	Particulate ≤ 1 microns	Input		[µg/m^3^]	PM_1_	0–249.38
8	Particulate ≤ 2.5 microns	Input		[µg/m^3^]	PM_2.5_	0–1040.64
9	Particulate ≤ 10 microns	Input		[µg/m^3^]	PM_10_	0–539.59
10	Time	Output		[h]	t	0–1176

**Fig. 2:**
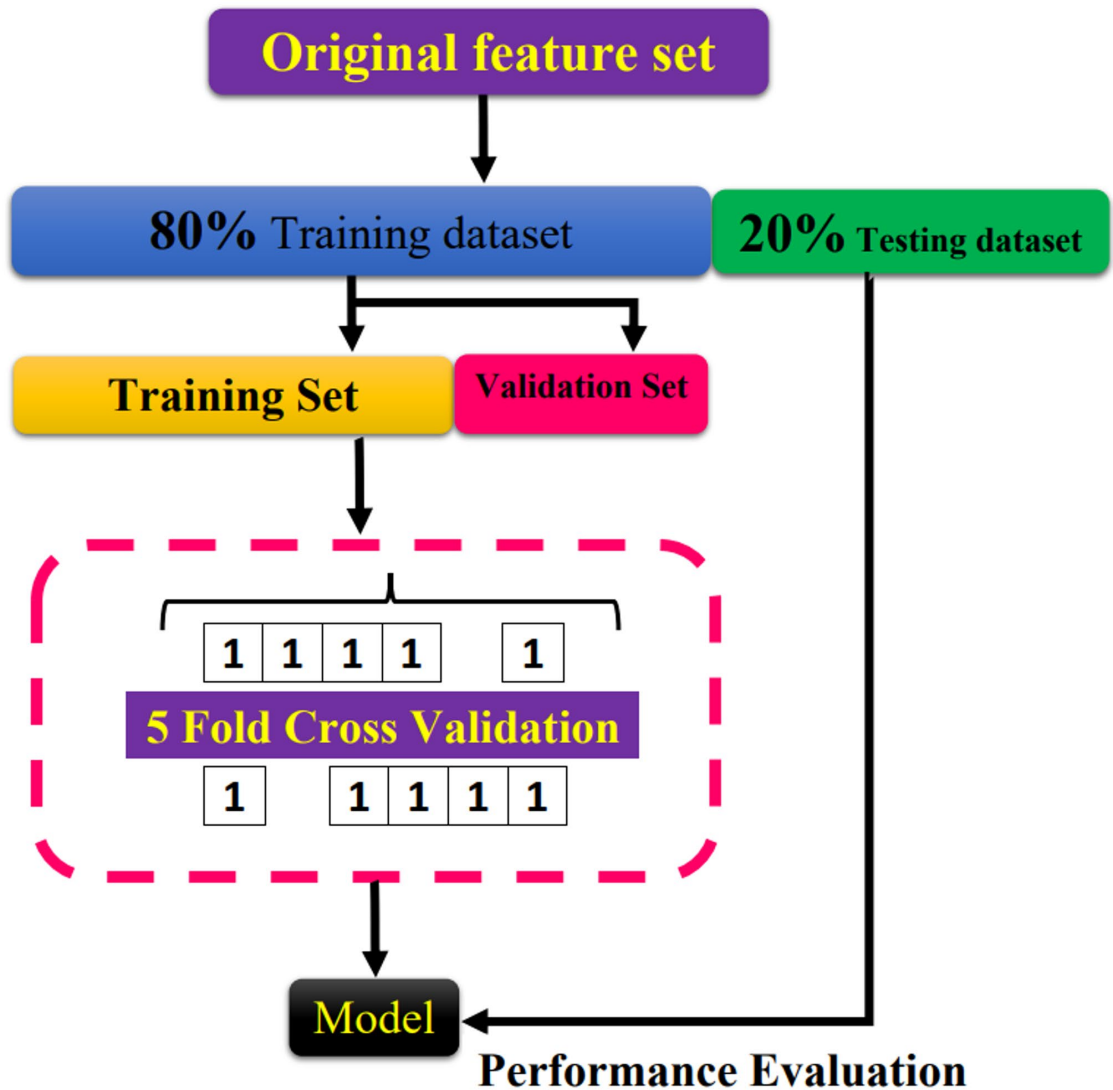
The schematic diagram of the K-fold cross-validation.

Considering the dataset contains 6.35 million samples, 5-fold cross-validation is efficient and practical to evaluate model performance. In fact, with such a large dataset, five-fold cross-validation ensures that the model sees different subsets for training and testing, providing a much more robust assessment of its generalization ability with less risk of overfitting. Thus, each fold would contain an approximate number of 1.27 million samples, which is a good balance between computational efficiency and model accuracy. Larger datasets might allow for more folds, say ten-fold cross-validation, but generally speaking, five-fold provides very reliable performance estimates without excessive computational costs. Moreover, this prevents bias because the model is tested on several portions of the data. This will ensure that the data is well-shuffled before splitting into folds that could contain some sort of ordering effects. Five-fold cross-validation has been chosen because it can catch the performance of a model really well while keeping computation time and resources usage reasonably low (Fig. 2 shows a diagram for five-fold).

### Error management and comparing the residuals and impact of hidden layers for air quality and environmental conditions in household air pollution

**Fig. 3:**
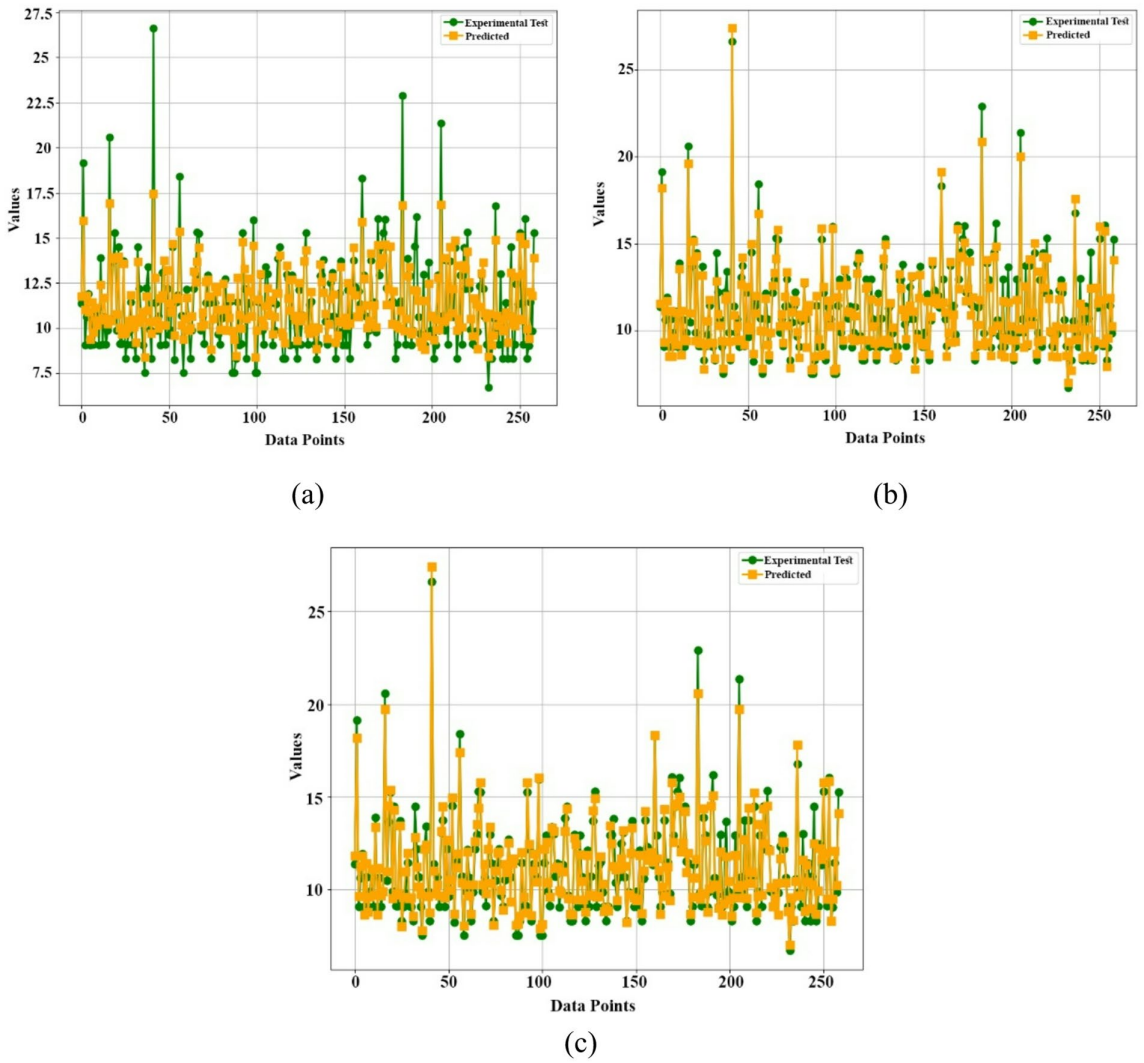
Improving ML model performance through training and validation error analysis: (**a**) GNN, (**b**) RNN, (**c**) EBM.

Monitoring air quality and environmental conditions in households is critical for assessing exposure risks and developing effective mitigation strategies^34,35^. ML models can offer valuable predictive insights and adaptive control mechanisms; however, their reliability hinges on minimizing training error while maintaining low validation error to ensure generalizability. A key challenge in this process is managing the trade-off between bias and variance. High bias can lead to underfitting, where the model fails to capture important patterns in the data. Conversely, high variance–often a sign of overfitting–occurs when a model learns the training data too well, including its noise and anomalies, leading to poor performance on unseen data. Overfitting results in a low training error but high validation error, rendering the model ineffective in real-world scenarios. In this study, we examine the interaction between training and validation errors across several ML models designed for household air quality monitoring, including EBM, RNN, and GNN. These models were trained using techniques such as k-fold cross-validation and hyperparameter tuning to control for overfitting and reduce bias. As shown in Fig. 3, the GNN model (Fig. 3a) demonstrates good generalization, with closely aligned training and validation losses, suggesting low variance and minimal overfitting. The RNN model (Fig. 3b) exhibits a low error rate, indicating stable learning and well-managed complexity. Similarly, the EBM model (Fig. 3c) maintains a consistent performance across training and validation phases, reflecting a balanced bias-variance trade-off.

**Fig. 4:**
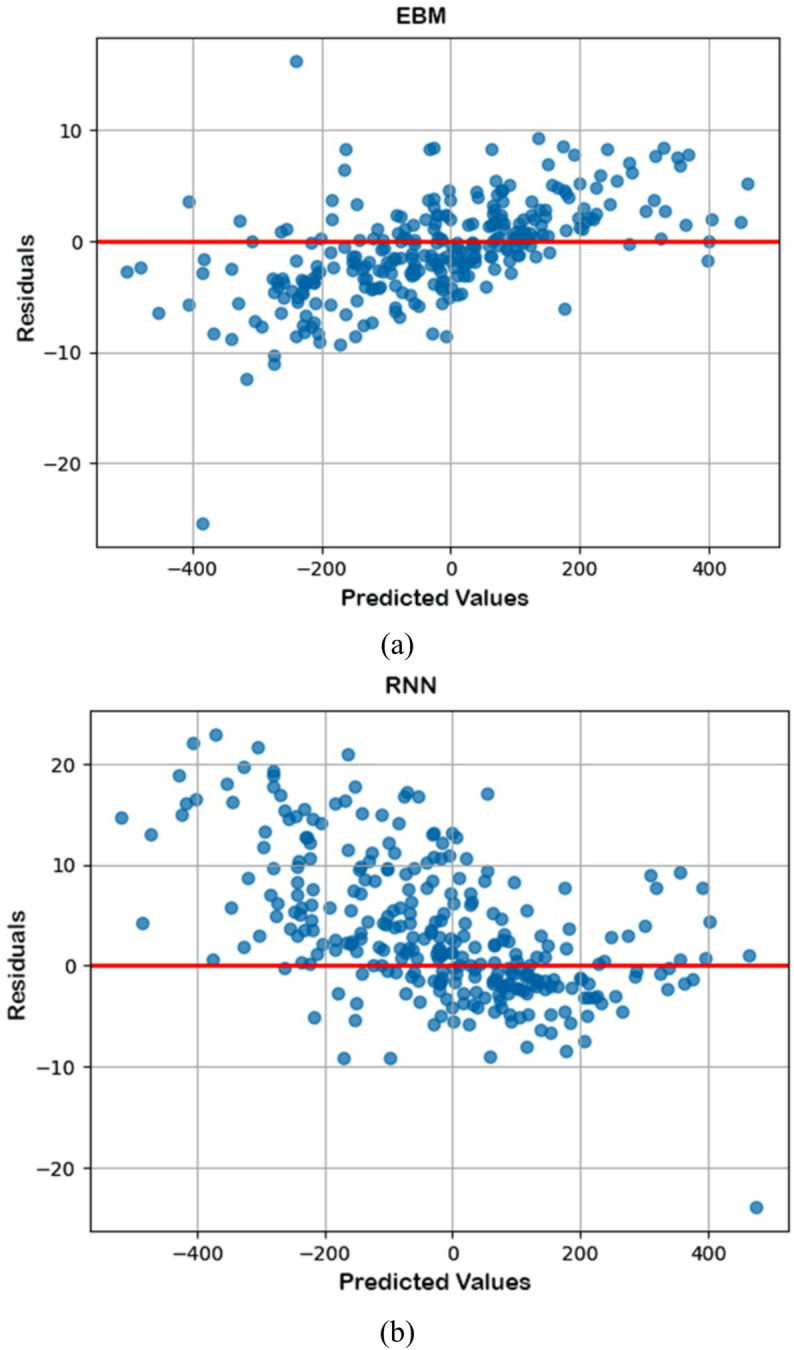

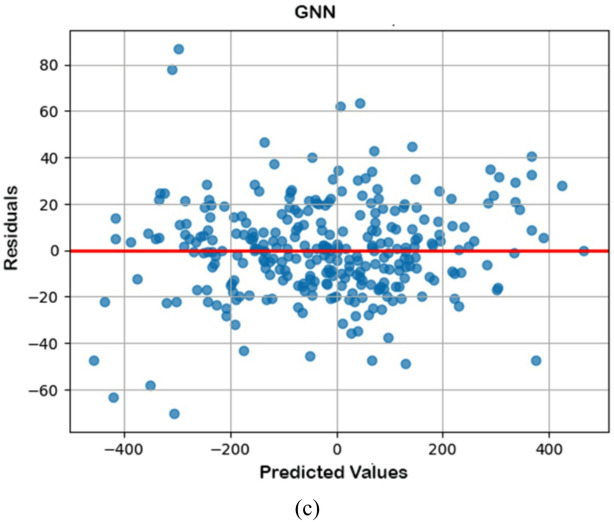
Comparison of residuals in ML models.

Among advanced machine learning models, GNN, EBM, and RNN each have distinct strengths in predicting air quality. GNNs are particularly effective at capturing spatial relationships and interactions among geographically distributed monitoring sites, making them well-suited for analyzing pollutant dispersion patterns. EBMs offer a strong balance between predictive accuracy and interpretability, providing clear insights into the factors that contribute to indoor air pollution. While GNNs excel at modeling structural dependencies, RNNs are designed to capture temporal dependencies and perform well in predicting time-series data, such as variations in pollutant concentrations caused by cooking or heating activities. Figure 4 compares the cumulative residuals of the three models, highlighting differences in their predictive performance. Although all models show residuals centered near zero, indicating generally accurate predictions, the variability and range of residuals reveal important distinctions. The residuals for EBMs are tightly clustered between − 10 and 10 (Fig. 4a), reflecting stable and consistent predictions with minimal deviation from actual values. This reliability and interpretability make EBMs well-suited for transparent decision-making. In contrast, the RNN residuals vary more widely, ranging from − 10 to 20 (Fig. 4b). This increased variability suggests that while RNNs effectively capture temporal trends, they face challenges in modeling complex sequential patterns consistently. GNNs show the greatest residual spread, between − 60 and 80 (Fig. 4c). Although GNNs are powerful in modeling complex spatial and nonlinear relationships, their sensitivity to outliers and structural changes in input data leads to higher prediction variability.

In the fast-evolving field of machine learning, hyperparameter optimization is a very important process that greatly influences the performance and generalization capabilities of predictive models^36,37^. Unlike model parameters, which are learned during the training process, hyperparameters are predefined settings that govern how the learning algorithm operates; they influence its efficiency and ability to generalize to unseen data^38^. This balance of model complexity is very important, with key hyperparameters being the learning rate, regularization strength, and maximum depth of decision trees, which all help avoid overfitting and underfitting to obtain robust and accurate predictions (Details are given in Table 3). In predictive modeling for air quality and environmental conditions, considering household air pollution, hyperparameter optimization has become a game-changing approach to improving the performance of state-of-the-art machine learning models, including Recurrent Neural Networks, Graph Neural Networks, and Explainable Boosting Machines. Among modern innovations, automatically performing hyperparameter optimizations, courtesy of advanced libraries including Optuna and Hyperopt, now empowers the whole process using sophisticated methods that include Bayesian optimization, Tree-structured Parzen Estimator (TPE), and early stopping criteria. Unlike traditional methods of hyperparameter tuning such as GridSearchCV, and RandomizedSearchCV, which rely on an exhaustive or random exploration of the hyperparameter space in a very computationally intensive way, Optuna and Hyperopt do adaptive search; hence, their computational resources are concentrated in the most promising regions of the hyperparameter space. For example, Optuna accelerates the optimization process by way of dynamic trial management and pruning strategies to discard poor trials as early as possible, whereas Hyperopt relies on the TPE algorithm to adaptively focus more on exploiting configurations that are likely to offer better improvement. These methods are particularly effective for optimizing critical hyperparameters, such as the number of layers and neurons in RNNs, feature subset depth in GNNs, and learning rate and boosting iterations in EBMs. It would therefore allow doing automatic hyperparameter tuning without manual and time-consuming tuning, defining the complete search space, and iteratively evaluating configurations by considering the performance metrics: MAE, RMSE, and R^2^ score.

**Table 3:** Enhancing machine learning performance in air quality and environmental modeling via automated hyperparameter optimization.

No.	Models	Hyperparameters	
1	*RNN*	Learning_rate	→ 0.0203
		Dropout rate	→ 0.4
		Batch size	→ 16
		Sequence length	→ 30
		Number of recurrent units	→ 195
		Optimizer	→ Adam
2	*GNN*	Hidden_channels	→ 52
		Num_classes	→ 1
		lr	→ 0.01
		Learning_rate	→ 0.029
		Pooling_type	→ sortpool
		Dropout	→ 0.2
		Activation	→ ReLU
		Batch_size	→ 32
3	*EBM*	Learning_rate	→ 0.1
		n_estimators	→ 100
		Max_depth	→ 3
		Min_samples_leaf	→ 2
		Early_stopping_rounds	→ 10
		Random_state	→ 42
		Max_bins	→ 150
		Max_interaction_bins	→ 145

## Results, discussion, and optimization

### Assessing land tenure, firewood collection, and cooking practices

Kericho County in Kenya is characterized by significant cultural and socio-economic diversity, shaped by a multi-ethnic population, varied economic activities, and a blend of traditional and modern lifestyles^39^. Despite this dynamic context, most households continue to rely on traditional cooking methods, particularly the three-stone stove, which produces high levels of smoke and contributes to respiratory and other health risks^40^. The heavy dependence on firewood as the primary cooking fuel also imposes economic strain on households, especially for those with limited access to land for fuel collection, while simultaneously raising environmental concerns. The questionnaire administered in this study comprised eight sections. Section A documented household identification details and survey administration procedures. Section B captured socioeconomic and demographic characteristics, including household composition, educational attainment, and income levels. Section C focused on cooking characteristics and patterns of fuel use. Section D examined the technical performance of, and user preferences for, traditional and improved cookstoves. Section E gathered detailed cooking information, including cooking location, types of food prepared, and cooking duration. Section F addressed institutional characteristics, such as group membership and access to information sources. Finanly, Section G explored financing and sustainability issues related to the adoption of improved cookstoves. Air quality monitoring was undertaken at both indoor and outdoor sampling locations to assess exposure differences associated with household cooking. Indoor measurements were conducted in the primary cooking area at approximately 1.2–1.5 m above floor level and within about 1 m of the stove, corresponding to the breathing zone of the cook. Outdoor measurements were obtained approximately 3–5 m from the kitchen structure to represent background environmental conditions. Pollutant concentrations were recorded at regular intervals during each monitoring session, covering both cooking and non-cooking periods in order to capture temporal variability. Missing or invalid data resulting from instrument malfunction, calibration drift, or power interruptions were identified, flagged, and excluded. Minor gaps were addressed through appropriate interpolation techniques, whereas datasets with substantial missing values were removed from the final statistical analysis. The survey covered a total of 1200 households. Following data screening and cleaning, 49 observations were excluded, resulting in a final analytical sample of 1151 households. The results presented in this study summarise the principal socioeconomic and demographic characteristics of the surveyed households. Indoor–outdoor (I/O) pollution ratios were not formally analysed because the primary objective was to examine short-term fluctuations in indoor pollutant concentrations driven by cooking behaviour rather than ambient infiltration. As cooking emissions dominated peak concentrations and outdoor pollution levels remained relatively low and stable during the monitoring period, I/O ratios were not considered to provide additional explanatory value. Indoor air movement or wind speed was not measured because the study dwellings lacked controlled mechanical ventilation, and indoor airflow patterns were minimal and highly variable, making reliable point measurements difficult. Furthermore, the study focused primarily on pollutant generation from cooking rather than dispersion modelling.

Findings from 1200 households, summarized in Table 4, show that wives are the primary decision-makers in 69.2% of households, while 15.3% of decisions are made jointly by spouses. Overall, women influence cooking-related choices in 84.5% of households, highlighting their central role and increased exposure to the hazards of biomass stoves. Demographic and socio-economic data indicate that most household heads (43.7%) are aged 36 to 50—a socially and economically active group well-suited for targeted clean energy interventions. Additionally, 77.8% of household heads are married, suggesting family stability that may support behavior change. Education levels vary: 42.2% have primary education, 32.9% secondary, and 17.8% tertiary education, all of which influence awareness and access to cleaner technologies. A small portion (3.56%) of households includes members with disabilities, representing a group with specific energy needs. Household sizes average five members (ranging from one to thirteen), with larger families requiring more cooking fuel, which increases emissions and health risks, particularly for women who spend more time cooking. Monthly incomes average Ksh 8,372.29 but vary widely. While some households can afford cleaner stoves, many are economically constrained to continue using inefficient methods. Agriculture remains the primary income source for 57.4% of households, reflecting the county’s rural nature. Because biomass fuel is readily available to these communities, improvements in agricultural productivity and market access may support economic growth and facilitate transitions to cleaner cooking technologies. Health issues linked to traditional cooking persist, with 14.8% of households reporting symptoms like eye irritation and redness from smoke exposure. Regarding firewood access, 58.6% of households lack land and rely on purchased or communal wood, while 41.4% own land and collect firewood freely, though both groups remain vulnerable to health and environmental risks. Land sizes vary widely (0.1 to 25 acres), averaging 1.5 acres, with significant disparities in land ownership. Among landowners, 60.2% hold freehold titles and 18.7% use communal land, indicating relatively secure tenure for many. Firewood consumption averages 16.6 bundles per household per week, costing about Ksh 181.27, reflecting the inefficiency and expense of traditional cooking fuels. Regarding stove types, 33.1% of households use improved three-stone stoves—more efficient than traditional models–while 32.9% continue with traditional stoves. Although improved stoves reduce emissions and fuel use, the ongoing reliance on outdated technologies perpetuates health, environmental, and financial burdens.

**Table 4:** Summary of survey findings on cooking practicesland tenure, and firewood usage.

Challenges	Finding
1. Decision-making on cooking methods and stoves	69.2% of households have wives as the primary decision-makers 15.3% report joint decision-making 14% have husbands as the sole decision-makers
2. Demographics and socio-economic characteristics	Most household heads (43.7%) are aged 36–50 years 77.8% are married, 11.8% are single, and 8.7% are widowed/widowers 42.2% completed primary school
3. Economic and health information	Average monthly household income is Ksh 8,372.29, with high income disparity 57.4% rely on farming for income 14.8% report health problems linked to cooking
4. Land ownership and firewood collection	58.6% do not own land for firewood collection Average land size among owners is 1.5 acres Freehold land ownership is the most common (60.2%)
5. Cooking and fuel usage	Average weekly firewood consumption: 16.6 bundles, costing Ksh 181.27 33.1% use Improved 3-Stone Stoves, 32.9% use Traditional Three-Stone Stoves Many households rely on external sources for firewood

### Design and performance evaluation of an improved cookstove during cold start, hot start, and simmering phases

The stove was designed using materials and fuels that are locally available, making it affordable and easy to adopt. Traditional cooking methods-such as open fires or basic stoves-can be harmful to health, especially for women and children, due to smoke and exposure to open flames. The study was carried out in Belgut Constituency, Kericho County, Kenya, where people mainly use charcoal and Eucalyptus grandis (rose gum) for cooking. These fuels were chosen because they are easy to find and have a high energy output. The stove was built with a combustion chamber made from refractory bricks that could withstand high temperatures. It was insulated with vermiculite to keep the heat inside and supported with hardcore, cement, and liners for strength. The stove measures 115 $\:\times\:$ 70 $\:\times\:$ 36 cm^3^, and it includes a chimney that is 15 cm in diameter and 2 m tall to help remove smoke and gases, improving indoor air quality. To test how well the stove performs, especially in terms of emissions, a Water Boiling Test (WBT) was used. This test measures the amount of carbon monoxide (CO), carbon dioxide (CO_2_), and particulate matter (PM_2.5_) released during use. The WBT has three phases: high-power cold start, high-power hot start, and simmering-each reflecting different cooking conditions that affect emissions.

**Table 5:** Key performance metrics of charcoal fuel in water boiling test across different phases.

Parameter	Units	High power (cold start)	High power (hot start)	Low power (simmer)
Time to boil (avg)	min	32	26	–
Temp-corrected time to boil	min	33	28	–
Burning rate	g/min	11	16	5.1
Thermal efficiency	%	19%	17%	37%
Specific fuel consumption	g/l	74	83	62
Temp-corrected specific consumption	g/l	76	88	–
Specific energy consumption	kJ/l	2265	2620	1838
Firepower	watts	5591	7733	2521
Turn down ratio	–	–	–	2.2
CO emissions	g/MJ	52.4	52.4	0.383
PM emissions	mg/MJ	528	528	4.0

The stove’s performance was evaluated across three phases of the Water Boiling Test (WBT): High Power Cold Start, High Power Hot Start, and Low Power (Simmer). Thermal efficiency was highest during the Simmer phase, reaching about 37%, indicating better heat conservation at lower power (As shown in Table 5). In contrast, both the Cold and Hot Start phases showed lower efficiencies (~ 17–19%), which is typical due to the rapid heating required. The burning rate was highest during the High Power Hot Start (~ 16 g/min) and Cold Start (~ 11 g/min), with the lowest rate observed in Simmer (~ 5 g/min), reflecting reduced combustion for lower heat demand. Specific fuel consumption was lowest during the Simmer phase (62 g/l), confirming efficient low-power operation. However, it peaked during the High Power Hot Start (88 g/l), likely due to incomplete combustion or heat loss. Emissions were highest during the Cold Start phase, with Carbon Monoxide (CO) emissions reaching ~ 123 g/MJ and Particulate Matter (PM) emissions at ~ 1172 mg/MJ. These emissions dropped substantially during the Hot Start phase (~ 5.2 g/MJ CO and ~ 231 mg/MJ PM) and were even lower during the Simmer phase (~ 0.38 g/(min L) CO and ~ 4.0 mg/(min L) PM). This highlights the stove’s higher emissions during ignition and cold operation, which can pose health risks. Firepower peaked at 5600 W during the Cold Start and 7700 W during the Hot Start, suitable for fast boiling, but it dropped to ~ 2500 W during Simmer, aligning with the reduced energy demand. The stove’s Turn Down Ratio, measuring its ability to adjust power levels, was approximately 2.22, indicating moderate flexibility. In comparison to benchmark values, the stove performed better in thermal efficiency and specific fuel consumption during the Simmer phase. However, CO and PM emissions during the Cold Start phase exceeded benchmark values. To improve performance, it is recommended to add a preheating phase or implement hot start strategies to reduce emissions during the Cold Start.

**Table 6:** Key performance metrics of eucalyptus grandis (rose gum, grand eucalyptus) fuel in water boiling test across different phases.

Parameter	Units	High power (cold start)	High power (hot start)	Low power (simmer)
Time to boil (avg)	min	17.33	16.33	–
Temp-corrected time to boil	min	18	17	–
Burning rate	g/min	37	36	15
Thermal efficiency	%	15.56	17.33	19.30
Specific fuel consumption	g/L	133	122	172
Temp-corrected specific consumption	g/L	138	130	–
Specific energy consumption	kJ/L	2549	2389	3166
Firepower	W	11,483	11,146	4489
Turn down ratio	–	–	–	3.0
CO emissions	g/MJ	43.7	4.8	–
PM emissions	mg/MJ	1947	385	–

Table 6 presents the key performance metrics of Eucalyptus Grandis fuel during the Water Boiling Test across different phases. The High Power (Cold Start) phase demonstrates the longest time to boil, averaging 17.33 min, with a thermal efficiency of 15.56%. This phase also shows high emissions, particularly in carbon monoxide (CO) at 43.7 g/MJ and particulate matter (PM) at 1947 mg/MJ, indicating less efficient combustion and higher pollution. In contrast, the High Power (Hot Start) phase achieves a faster boiling time of 16.33 min, with improved thermal efficiency at 17.33%. CO emissions are significantly reduced to 4.8 g/MJ, and PM emissions drop to 385 mg/MJ, indicating better combustion after the stove reaches its operating temperature. During the Low Power (Simmer) phase, the stove exhibits the highest thermal efficiency of 19.30%, although it has a lower firepower of 4,489 W. This phase is associated with the highest specific energy consumption (3166 kJ/L) and specific fuel consumption (172 g/L), reflecting the longer cooking times at reduced power. The turn-down ratio for this phase is moderate at 3.0, suggesting flexibility in adjusting the heat level. Overall, the stove operates most efficiently during the High Power (Hot Start) phase in terms of both fuel consumption and emissions, while the Cold Start phase shows room for improvement in combustion efficiency and emissions control.

### Investigating performance metrics and comparing training time in ML models

This study compares the performance of three ML models, namely EBM, RNN, and GNN, through comparison using six key statistical measures of R^2^, MAE, EVS, SMAPE (%), MSE, and RMSE to yield a holistic view of a model’s predictability, robustness, and generalization ability on different datasets. Amongst the models studied, EBM performed the best for all other models with an R^2^ of 0.9498 and MAE of 0.4757 for complex data patterns; similarly, RNN was a strong performer with an R^2^ of 0.9356 and MAE of 0.5419. GNN, which is powerful in other applications, has shown low accuracy, with an R^2^ of 0.9086 and high prediction errors with MAE of 0.5606 and RMSE of 0.8244 (Table 7 presents the comparative performance of the models based on these metrics). It follows that through the comparative analysis above, both EBM and RNN become effective in optimizing air quality and environmental conditions, with EBM as the most accurate and reliable model. It therefore has a particular suitability in applications requiring predictions with high precision and consistency. Moreover, EBM provides great value in terms of explainability, with interpretable results being obtained from EBM-which is a necessity for most applications needing some form of transparency in decision-making.

**Table 7:** Performance metrics of the Ml models for air quality and environmental conditions in household air pollution.

Metric/algorithm	EBM	RNN	GNN
MAE	0.4757	0.5419	0.5606
RMSE	0.6107	0.6922	0.8244
MSE	0.3730	0.4792	0.6796
R^2^-value	0.9498	0.9356	0.9086
EVS	0.9512	0.9371	0.9102
SMAPE (%)	8.74	9.92	11.35

### Key contributing factors in household air pollution caused by incomplete biomass combustion

**Fig. 5:**
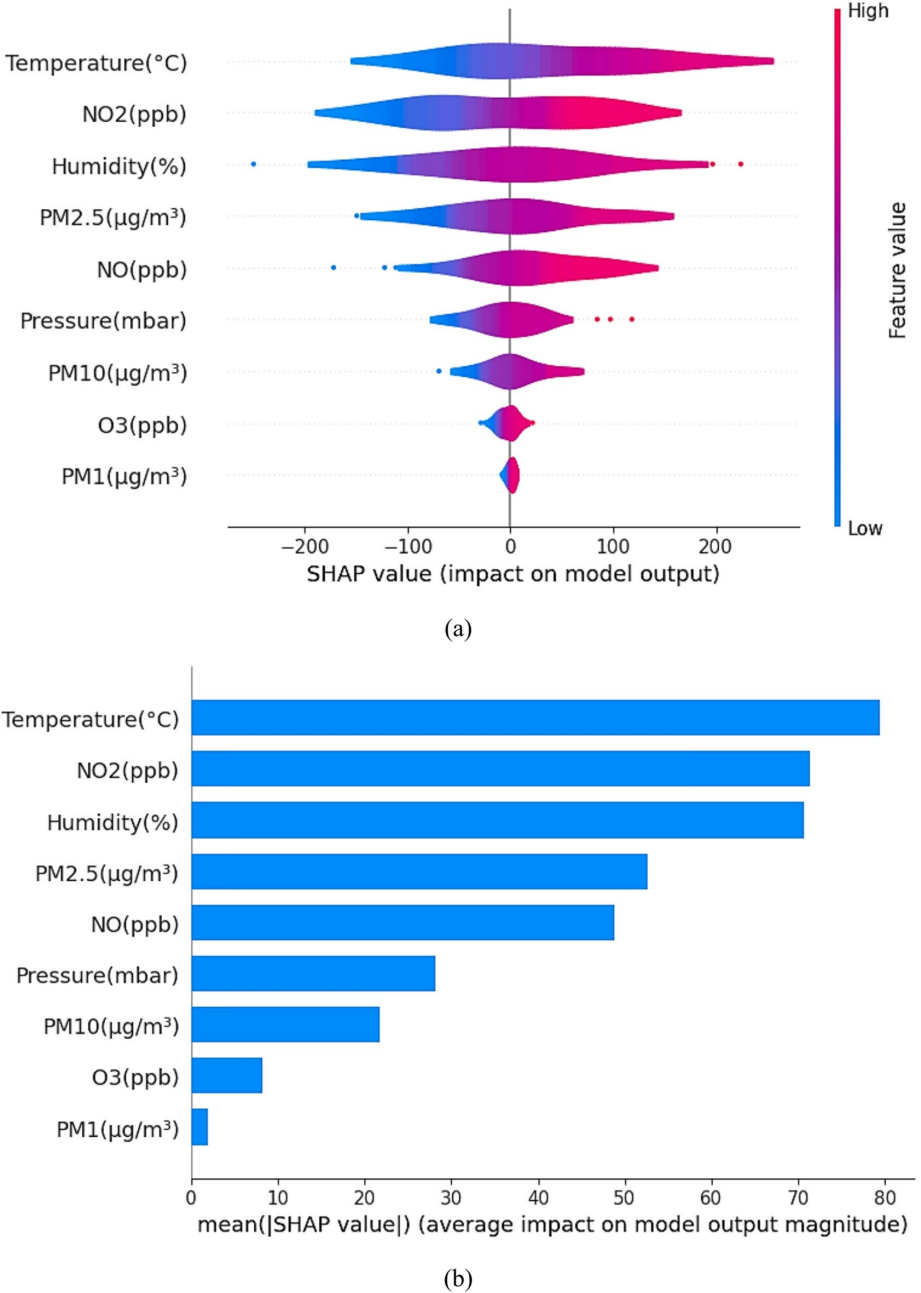
SHAP analysis and ML models for investigating household air pollution from incomplete biomass combustion.

This study investigates household air quality using the EBM combined with Tree SHAP analysis to uncover key drivers of indoor pollution. Temperature emerged as the dominant factor, primarily due to its influence on combustion efficiency: elevated temperatures promote more complete combustion, reducing CO and PM, whereas lower temperatures lead to incomplete burning and increased emissions (see Fig. 5a). NO_2_ ranked second in importance. It forms under high-temperature combustion and often accumulates indoors due to inadequate ventilation, contributing to the formation of secondary pollutants and posing serious health risks. Humidity followed as the third most impactful factor, as it influences PM dynamics, combustion behavior, and chemical interactions in indoor air. High humidity not only increases particle size and toxicity but also exacerbates emissions from damp biomass fuels. Fine particulate matter (PM_2.5_) also played a critical role due to its capacity to penetrate deep into the respiratory system and bloodstream. Its presence was often intensified by inefficient stove designs and poor ventilation (see Fig. 5b). Additional contributors included NO, PM_10_, PM_1_, and ozone. NO can form even at lower combustion temperatures and is a precursor to NO₂. Coarse and ultrafine particles (PM_10_ and PM_1_) originate from biomass burning and are linked to respiratory and cardiovascular disorders. Ozone, a secondary pollutant, can infiltrate indoor spaces and react with VOCs, compounding adverse health effects. Finally, atmospheric pressure influenced combustion conditions and pollutant dispersion, particularly at high altitudes where reduced oxygen levels alter fuel behavior.

### Feature sensitivity and model behavior via PDPs

**Fig. 6:**
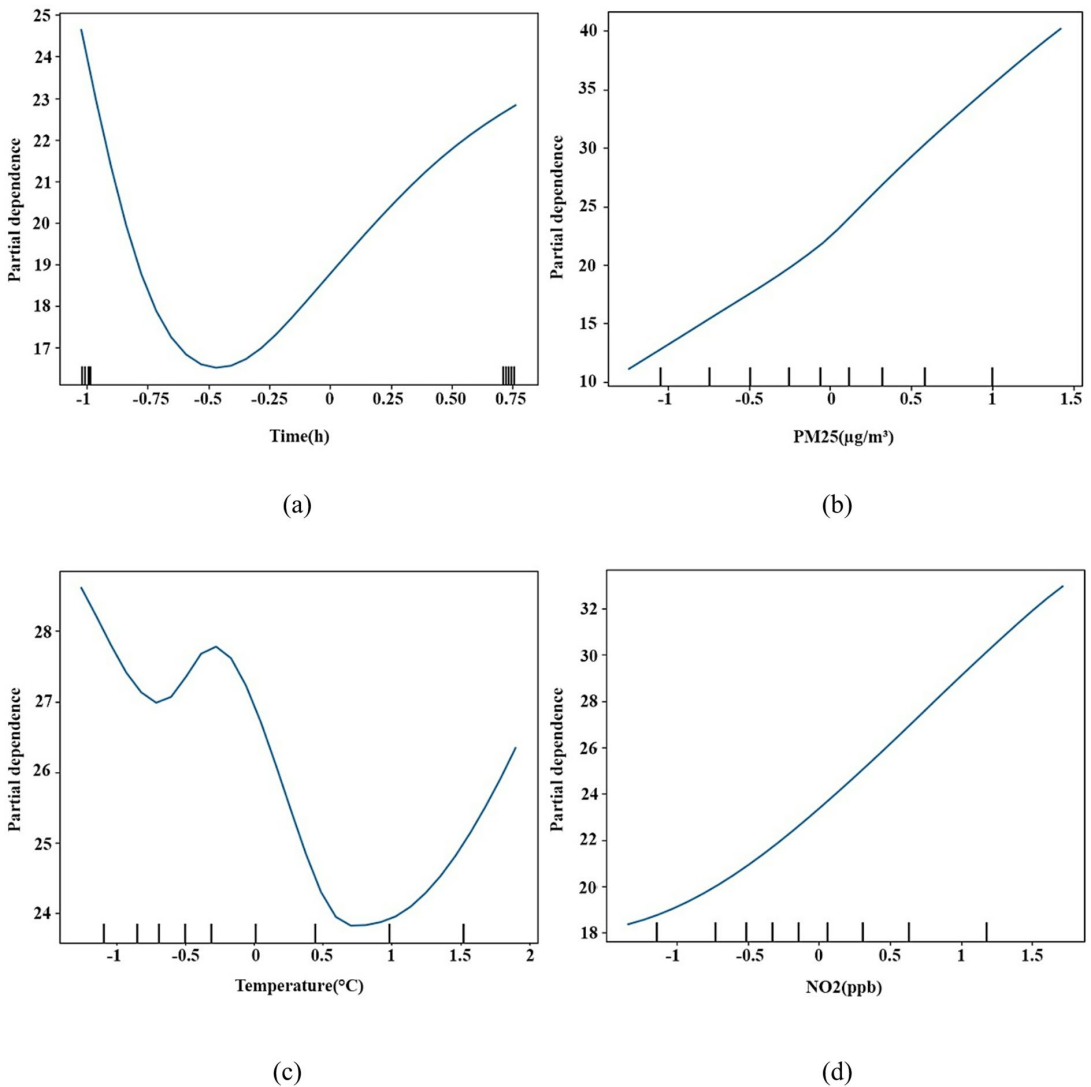
PDP analysis of Time, PM_2.5_, Temperature, and Nitrogen dioxide.

Figure 6 presents a series of PDPs generated using the EBM model to assess the isolated effects of four key variables–Time, PM_2.5_ concentration, Temperature, and NO_2_–on the predicted levels of household air pollution resulting from biomass combustion. These plots illustrate the marginal influence of each feature on the model’s output, with all other variables held constant, thereby facilitating a more interpretable understanding of the model’s internal decision-making process. Figure 6a displays the relationship between normalized Time (in hours) and the partial dependence of the predicted pollution levels. The curve exhibits a distinct U-shaped pattern, beginning at a relatively high value (~ 25) when Time = -1, declining to a minimum (~ 17) around Time = − 0.5, and then gradually increasing to approximately 23 at Time = 0.75. This trend suggests that pollution levels are generally lower around midday and higher during the early morning and late evening hours, likely corresponding to periods of intensified indoor activities such as cooking or heating. Figure 6b depicts the effect of PM_2.5_ (particulate matter with a diameter ≤ 2.5 μm) concentration on the model output, demonstrating a strong positive linear relationship. As PM_2.5_ levels increase from − 1.5 to 1.5, the partial dependence rises steadily from approximately 10 to 40, indicating that elevated PM_2.5_ concentrations significantly heighten the predicted indoor pollution burden. This underscores the prominent role of fine particulate matter as a major contributor to indoor air quality deterioration in biomass-using households. Figure 6c explores the influence of Temperature on predicted pollution, revealing a markedly non-linear and oscillatory relationship. The partial dependence initially peaks at around 29 when Temperature = − 1.5 °C, then slightly declines, increases near − 0.5 °C, drops to a pronounced minimum (~ 24) at approximately 0.25 °C, and subsequently rises again with increasing temperature. This complex pattern implies that temperature affects pollution predictions in a nuanced manner, potentially due to variations in combustion efficiency, air exchange rates, or seasonal biomass usage behaviors. Lastly, Fig. 6d illustrates the effect of NO_2_ concentration, which exhibits a clear monotonic increasing trend. The partial dependence increases from roughly 18 to over 32 as NO_2_ ranges from − 1.5 to 1.5, indicating a strong and direct positive relationship. This finding highlights the importance of nitrogen dioxide–commonly produced through incomplete combustion–as a robust indicator of deteriorating indoor air quality. Together, these PDPs provide valuable insights into the individual contributions of each feature to the model’s predictions, enhancing interpretability and supporting data-driven strategies for mitigating household air pollution.

### Discussion

The findings confirm the continued dominance of traditional biomass cooking in rural households, consistent with previous studies across sub-Saharan Africa that identify the three-stone stove as a major contributor to indoor air pollution and household health risks. The strong influence of women in cooking decisions aligns with existing literature showing that women are both primary energy managers and the most affected by household air pollution exposure. The relatively high proportion of household heads aged 36–50 suggests an opportunity for targeted clean cooking interventions, as this economically active group may be more receptive to adopting improved technologies. Education levels also play an important role, as higher educational attainment is generally associated with greater awareness and willingness to adopt cleaner energy solutions, a trend observed in earlier energy transition studies. Despite the presence of improved cookstoves in some households, their adoption remains moderate. This indicates persistent barriers such as affordability constraints, fuel accessibility, and cultural preferences for traditional cooking methods. Similar findings have been reported in previous research, where improved stove adoption does not always translate into exclusive use, resulting in continued exposure to biomass smoke. Economic limitations remain a major constraint. Although some households can afford cleaner stoves, many continue using traditional biomass due to low and unstable incomes. The reliance on agriculture as the primary livelihood reinforces dependence on firewood, which is often readily available but inefficient and environmentally unsustainable. However, improvements in agricultural productivity and market access could enhance household incomes and facilitate transitions toward cleaner energy. Health symptoms reported by households further highlight the ongoing burden of household air pollution. Although the reported prevalence appears moderate, it likely underestimates the full health impact because many respiratory effects develop gradually and may not be immediately recognized by households.

## Conclusions

This study highlights the effectiveness of integrating empirical field data with advanced machine-learning and statistical approaches to better understand and mitigate household air pollution caused by incomplete biomass combustion. The combined use of explainable boosting machines, recurrent neural networks, and graph neural networks enabled a comprehensive analysis of the complex relationships among indoor and outdoor air-quality parameters, environmental conditions, and combustion behavior, while also improving prediction reliability and interpretability.

Survey findings provide important socio-economic and behavioral context, showing that household structure, education level, income sources, and culturally influenced decision-making, particularly the central role of women in cooking choices, strongly affect cooking practices and technology adoption. Continued reliance on traditional biomass stoves, along with reported smoke-related health symptoms, underscores the persistent exposure risks and the need for accessible and culturally appropriate clean-cooking solutions.

Experimental evaluation demonstrates that stove performance varies significantly across operational phases, with more stable combustion generally associated with improved efficiency and reduced emissions, while ignition phases contribute disproportionately to pollutant release. Environmental factors, particularly temperature, humidity, and fine particulate matter, were identified as key drivers influencing combustion quality, pollutant formation, and dispersion patterns. The modelling analysis further confirms the importance of particulate and nitrogen-related pollutants in shaping household air-pollution dynamics and highlights the value of interpretable machine-learning tools for identifying dominant predictors and supporting targeted interventions.

### Implications for policy and research

The findings suggest that promoting clean cooking transitions in rural settings requires integrated approaches addressing affordability, awareness, and fuel accessibility. Women should remain central to intervention strategies due to their dominant role in household cooking decisions. Future research should incorporate longitudinal monitoring, detailed ventilation assessment, and objective health measurements to better quantify exposure and long-term impacts.

### Advantages of the study

This study provides a large household sample and integrates socio-economic, behavioural, and environmental data. The combination of survey data and real-time air quality monitoring strengthens the reliability of the findings. Additionally, focusing on cooking-related pollution peaks provides valuable insight into short-term exposure patterns, which are often overlooked in household energy studies.

## Data Availability

The data that supports the findings of this study is available upon request. Please contact [Seyed Hamed Godasiaei and Obuks A. Ejohwomu] for access to the data.

## References

[1] Odame, M. L. & Adjei-Mantey, K. Household air pollution could make children grow shorter in sub-Saharan Africa; but can households help stem the tide on their own? *World Dev. Perspect.* **33**, 100562 (2024).

[2] Air pollution and indoor work efficiency. Evidence from professional basketball players in China. *J. Clean. Prod.* **399**, 136644 (2023).

[3] Wernecke, B., Langerman, K. E., Howard, A. I. & Wright, C. Y. Fuel switching and energy stacking in low-income households in South Africa: A review with recommendations for household air pollution exposure research. *Energy Res. Soc. Sci.* **109**, 103415 (2024).

[4] Nazli, S. N. et al. Indoor air quality: Bibliometric analysis of the published literature between 2018 and 2023. *Environ. Qual. Manag.* **34**, (2024).

[5] Golkarfard, V. & Talebizadeh, P. Numerical comparison of airborne particles deposition and dispersion in radiator and floor heating systems. *Adv. Powder Technol.* **25**, 389–397 (2014).

[6] Ochieng, C., Vardoulakis, S. & Tonne, C. Household air pollution following replacement of traditional open fire with an improved rocket type cookstove. *Sci. Total Environ.* **580**, 440–447 (2017). 28040224 10.1016/j.scitotenv.2016.10.233

[7] Pender, K., Romoli, F., Rodes, M., Fuller, F. A., Zeolla, M. & J. & Future strategies for decarbonisation of carbon fibre products: A roadmap to net zero 2050. *J. Clean. Prod.* **486**, 144525 (2025).

[8] Zhang, Q., Iqbal, S. & Shahzad, F. Role of environmental, social, and governance (ESG) investment and natural capital stocks in achieving net-zero carbon emission. *J. Clean. Prod.* **478**, 143919 (2024).

[9] Younger, A., Alkon, A., Harknett, K., Jean Louis, R. & Thompson, L. M. Adverse birth outcomes associated with household air pollution from unclean cooking fuels in low- and middle-income countries: A systematic review. *Environ. Res.* **204**, 112274 (2022). 34710435 10.1016/j.envres.2021.112274 PMC13719184

[10] Jerneck, A. & Olsson, L. A smoke-free kitchen: Initiating community based co-production for cleaner cooking and cuts in carbon emissions. *J. Clean. Prod.* **60**, 208–215 (2013).

[11] Younger, A. et al. Effects of a liquefied petroleum gas stove intervention on stillbirth, congenital anomalies and neonatal mortality: A multi-country household air pollution intervention network trial. *Environ. Pollut*. **345**, 123414 (2024). 38286258 10.1016/j.envpol.2024.123414 PMC13296888

[12] Qiu, A. Y., Leng, S., McCormack, M., Peden, D. B. & Sood, A. Lung effects of household air pollution. *J. Allergy Clin. Immunol. Pract.* **10**, 2807–2819 (2022). 36064186 10.1016/j.jaip.2022.08.031

[13] Benka-Coker, M. L. et al. Impact of the wood-burning Justa cookstove on fine particulate matter exposure: A stepped-wedge randomized trial in rural Honduras. *Sci. Total Environ.* **767**, 144369 (2021). 33429278 10.1016/j.scitotenv.2020.144369 PMC7919923

[14] Shupler, M. et al. Household concentrations and female and child exposures to air pollution in peri-urban sub-Saharan Africa: measurements from the CLEAN-Air(Africa) study. *Lancet Planet. Health*. **8**, e95–e107 (2024). 38331535 10.1016/S2542-5196(23)00272-3 PMC10864747

[15] Wassie, Y. T. & Adaramola, M. S. Analysis of potential fuel savings, economic and environmental effects of improved biomass cookstoves in rural Ethiopia. *J. Clean. Prod.* **280**, 124700 (2021).

[16] Phillip, E. et al. Improved cookstoves to reduce household air pollution exposure in sub-Saharan Africa: A scoping review of intervention studies. *PLoS One*. **18** (2023). 10.1371/journal.pone.0284908 PMC10138283 37104469

[17] Chen, N., Ma, L. L., Zhang, Y. & Yan, Y. X. Association of household solid fuel use and long-term exposure to ambient air pollution with estimated 10-year high cardiovascular disease risk among postmenopausal women. *Environ. Pollut*. **342**, 123091 (2024). 38061434 10.1016/j.envpol.2023.123091

[18] Li, C., Zhou, Y. & Ding, L. Effects of long-term household air pollution exposure from solid fuel use on depression: Evidence from national longitudinal surveys from 2011 to 2018. *Environ. Pollut*. **283**, 117350 (2021). 34034020 10.1016/j.envpol.2021.117350

[19] Ahmed, M., Shuai, C., Abbas, K., Rehman, F. U. & Khoso, W. M. Investigating health impacts of household air pollution on woman’s pregnancy and sterilization: Empirical evidence from Pakistan, India, and Bangladesh. *Energy* **247**, 123562 (2022).

[20] Ali, M. U. et al. Health impacts of indoor air pollution from household solid fuel on children and women. *J. Hazard. Mater* **416**, (2021). 10.1016/j.jhazmat.2021.126127 34492921

[21] Men, Y. et al. Mitigating household air pollution exposure through kitchen renovation. *Environ. Sci. Ecotechnol*. **23**, 100501 (2025). 39553849 10.1016/j.ese.2024.100501 PMC11565419

[22] Rao, N. D., Kiesewetter, G., Min, J., Pachauri, S. & Wagner, F. Household contributions to and impacts from air pollution in India. *Nat. Sustain.* **4**, 859–867 (2021).

[23] Ranzani, O. T. et al. Association of ambient and household air pollution with lung function in young adults in an peri-urban area of South-India: A cross-sectional study. *Environ. Int.* **165**, 107290 (2022). 35594814 10.1016/j.envint.2022.107290

[24] Scussiatto, O. Air pollution is associated with increased incidence-rate of head and neck cancers: A nationally representative ecological study. *Oral Oncol.* **150**, 106691 (2024). 38266316 10.1016/j.oraloncology.2024.106691

[25] Roy, A. & Acharya, P. Energy inequality and air pollution nexus in India. *Sci. Total Environ.* **876**, 162805 (2023). 36907412 10.1016/j.scitotenv.2023.162805

[26] Archer-Nicholls, S. et al. The regional impacts of cooking and heating emissions on ambient air quality and disease burden in China. *Environ. Sci. Technol.* **50**, 9416–9423 (2016). 27479733 10.1021/acs.est.6b02533

[27] Wang, L. et al. Role of Chinese cooking emissions on ambient air quality and human health. *Sci. Total Environ.* **589**, 173–181 (2017). 28262368 10.1016/j.scitotenv.2017.02.124

[28] Nduka, E. & Jimoh, M. Cooking energy, health, and happiness of women in Nigeria. *Energy J.* **45**, 79–106 (2024).

[29] Chen, J. et al. Global, regional, and national burden of cancers attributable to particulate matter pollution from 1990 to 2019 and projection to 2050: Worsening or improving? *J. Hazard. Mater.* **477**, 135319 (2024). 39059291 10.1016/j.jhazmat.2024.135319

[30] Zang, X., Feng, J. & Song, M. The impact of air pollution on household vulnerability to poverty: An empirical study from household data in China. *Econ. Anal. Policy*. **82**, 1369–1383 (2024).

[31] Kuye, A. & Kumar, P. A review of the physicochemical characteristics of ultrafine particle emissions from domestic solid fuel combustion during cooking and heating. *Sci. Total Environ.* **886**, 163747 (2023). 37146811 10.1016/j.scitotenv.2023.163747

[32] Saha, S., Das, P., Das, T., Das, P. & Roy, T. B. A study about the impact of indoor air pollution on cognitive function among middle-aged and older adult people in India. *Arch. Public. Health*. **82**, 1–11 (2024). 38664719 10.1186/s13690-024-01286-5 PMC11044570

[33] Baharane, V. & Shatalov, A. B. Assessment of the health impacts of air pollution exposure in East African countries. *Environ. Monit. Assess.* **196**, 1–13 (2024). 10.1007/s10661-024-12588-0 38565772

[34] Zeng, Y., Chen, J., Jin, N., Jin, X. & Du, Y. Air quality forecasting with hybrid LSTM and extended stationary wavelet transform. *Build. Environ.* **213**, 108822 (2022).

[35] Gilik, A., Ogrenci, A. S. & Ozmen, A. Air quality prediction using CNN+LSTM-based hybrid deep learning architecture. *Environ. Sci. Pollut. Res.* **29**, 11920–11938 (2022). 10.1007/s11356-021-16227-w 34554404

[36] Godasiaei, S. H. Predicting ash accumulation in industrial systems using machine learning: Enhancing maintenance and operational efficiency. *Particuology* **103**, 41–54 (2025).

[37] Dosumu, A., Godasiaei, S. H., Ejohwomu, O. A. & Mohandes, S. R. Generative physics-informed machine learning for modeling indoor air quality and its impact on student health and performance. *J. Environ. Manag.* **395**, 127676 (2025). 10.1016/j.jenvman.2025.127676 41135405

[38] Godasiaei, S. H., Talebizadehsardari, P. & Keshmiri, A. ML-Driven optimization of two-phase microfluidic cooling using acoustofluidic bubble actuation and nanoarray-coated micropin structures. *Sci. Rep.* **15**, 1–19 (2025). 41249281 10.1038/s41598-025-23871-6 PMC12624137

[39] Mbandi, A. M. et al. Assessment of the impact of road transport policies on air pollution and greenhouse gas emissions in Kenya. *Energy Strateg Rev.* **49**, 101120 (2023).

[40] Abok, F. A. et al. Model-derived characterization of particulate matter (PM2.5) and its species over Kenya during 1980–2020. *Open. Access. Libr. J.* **11**, 1–17 (2024).

